# Homeostasis in large networks of neurons through the Ising model - do higher order interactions matter?

**DOI:** 10.1186/1471-2202-14-S1-P166

**Published:** 2013-07-08

**Authors:** Dagmara Panas, Alessandro Maccione, Luca Berdondini, Matthias H Hennig

**Affiliations:** 1 Institute for Adaptive and Neural Computation, The University of Edinburgh, Edinburgh, EH8 9AB, UK; 2 Department of Neuroscience and Brain Technologies, Italian Institute of Technology, 16163 Genova, Italy

## 

Homeostatic activity in large networks of neurons is a relatively scantly explored area of neuroscience, both on experimental and computational level [1]. With recent advance in recording techniques, the lack of experimental data is gradually ceasing to be the limitation. New multielectrode arrays (MEA) allow for monitoring cultures of thousands of neurons over many days with high spatial resolution [2]. However, the interpretation of multi-neuron recordings is not straightforward and requires methods going beyond the simplest descriptive statistics.

Here we explore a novel approach to analyzing multi-unit neuronal activity recorded over a five day homeostatic experiment by employing the Ising model [3,4]. This statistical model explains the probability of multi-neuron spike patterns solely on the basis of firing rates and correlations, assuming an otherwise minimally structured distribution. Its application to a variety of recordings has helped re-evaluate the importance of neural interactions in shaping the global activity [3,4]. In addition, due to the models minimal structure, the quality of the fits can be treated as an indicator of higher-order interactions in the activity [4].

We compare the Ising model fits in the same preparation over several recordings: before, during and after CNQX application. We find that, in addition to the changes in firing rates and correlations, also the quality of the fits changes significantly across recordings (Figure 1). However, while firing rates and correlations to not appear to stabilize to a baseline level, the quality of the model fit does (Figure 1). Altogether this indicates that changes to first and second order statistics cannot explain the homeostatic changes in activity; and that higher order interactions might be a significant component of homeostatic compensation. Whether homeostatic maintenance of a complex higher-order dynamics is an effect of interplay of simple mechanisms or a global homeostatic set-point remains to be investigated.

**Figure 1:**
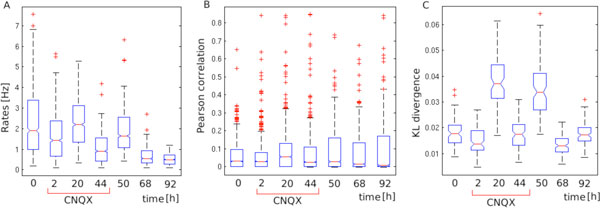
**Boxplot comparisons of different measures across seven recordings from the experimental culture**. **A **Firing rates; **B **Pearson correlation coefficient; **C **Kullback-Leibler divergence between the distributions of patterns predicted by the Ising model and the distributions of patterns observed in the data.

## References

[B1] Turrigiano G Too Many Cooks? Intrinsic and Synaptic Homeostatic Mechanisms in Cortical Circuit Refinement Annu Rev Neuroscience 2011 34 89 103 10.1146/annurev-neuro-060909-153238 21438687

[B2] Berdondini L Imfeld K Maccione A Tedesco M Neukom S Koudelka-Hep M Martinoia S Active pixel sensor array for high spatio-temporal resolution electrophysiological recordings from single cell to large scale neuronal networks Lab Chip 2009 9 2644 2651 10.1039/b907394a 19704979

[B3] Schneidman E Bialek W Ising models for networks of real neurons Nature 2006 440 1007 1012 10.1038/nature04701 16625187 PMC1785327

[B4] Ohiorhenuan IE Mechler F Purpura KP Schmid AM Hu Q Victor JD Sparse coding and high-order correlations in fine-scale cortical networks Nature 2010 466 617 621 20601940 10.1038/nature09178 PMC2912961

